# Economic Evaluation Methodologies of Remote Patient Monitoring for Chronic Conditions: Scoping Review

**DOI:** 10.2196/71565

**Published:** 2025-07-04

**Authors:** Siri Bjorvig, Elin Breivik, Jordi Piera-Jiménez, Carme Carrion

**Affiliations:** 1 Norwegian Centre for E-health Research, University Hospital of North Norway, Sykehusvegen 23, Tromsø, 9019, Norway, 47 97164725; 2 eHealth Lab Research Group, eHealth Center & School of Health Sciences, Universitat Oberta de Catalunya, Barcelona, Spain; 3 Catalan Health Service, Barcelona, Spain; 4 Digitalization for the Sustainability of the Healthcare System (DS3), Barcelona, Spain

**Keywords:** digital health, eHealth, telemonitoring, health economics, CHEERS guidelines, cost-effectiveness, budget impact analysis, cost methodology, Consolidated Health Economic Evaluation Reporting Standards

## Abstract

**Background:**

Remote patient monitoring (RPM) offers a potential solution to manage the increasing prevalence of chronic condition challenges in health care systems worldwide, but its economic evaluation remains challenging.

**Objective:**

This scoping review aimed to explore the methodologies used in economic evaluations of RPM interventions for chronic conditions, with a particular focus on cost identification, measurement and valuation, and reporting quality.

**Methods:**

A scoping review was conducted following the Joanna Briggs Institute methodology for scoping reviews. Systematic searches were carried out in Embase, MEDLINE, CINAHL, and Web of Science in week 40 of 2023, with no restrictions on the start date. No geographical restrictions were applied beyond requiring English-language publications. Studies were included if they reported a full or partial economic evaluation of an RPM intervention targeting patients with one or more chronic conditions. Screening and selection were performed independently by 2 reviewers. A total of 5473 records were identified, of which, 41 records met inclusion criteria after screening. Data were synthesized into key themes: study characteristics (design, population, setting), economic evaluation methods (types of analysis, comparator, perspectives, and outcome measures), cost estimation (identification, measurement, valuation), and adherence to the CHEERS (Consolidated Health Economic Evaluation Reporting Standards) 2022. Discrepancies were resolved through discussion. The review protocol was registered in the Open Science Framework.

**Results:**

A total of 41 papers, representing 40 studies, were included in the final review. Studies used diverse evaluation methods, such as cost-effectiveness analysis (20 studies), within which, 13 studies specifically conducted cost-utility analysis. Other approaches included cost-consequence analysis (7 studies), cost-minimization analysis (3 studies), cost-benefit analysis (2 studies), cost analysis (8 studies), and budget impact analysis (1 study). Cost estimation approaches varied across studies, with differences in cost identification, measurement, and valuation. Cost estimation methodologies varied, both in terms of which cost components were included and how costs were identified, measured, and valued. Commonly reported costs related to health care resource use and technology, but the data sources used, and the level of transparency provided, varied. Studies reported a range of outcome measures, including quality-adjusted life years, mortality, and financial indicators. Some studies reported multiple outcomes. Reporting inconsistencies were observed, and adherence to updated CHEERS 2022 standards was limited, particularly in sensitivity analyses and cost data transparency.

**Conclusions:**

This review highlights the diversity and methodological variability in economic evaluations of RPM interventions for chronic conditions. Key limitations include inconsistent cost methodologies and inadequate adherence to reporting standards, complicating cross-study comparisons. Future research should adopt more standardized, transparent reporting protocols to improve the reliability and utility of economic evidence for decision-makers considering RPM implementation.

## Introduction

The rising prevalence of chronic conditions, including cardiovascular diseases (CVD), cancers, chronic respiratory diseases (CRD), and diabetes mellitus (DM), represent a significant global health concern, affecting millions of individuals worldwide [1]. In many countries, the rise of these conditions has led to a strain on health care resources and personnel [2]. Concurrently, demographic shifts, such as a declining working-age population and an increase in the number of older individuals, further challenge the capacity of health care systems [3,4]. Managing chronic conditions while addressing workforce shortage has thus become a critical societal challenge.

Digital health interventions (DHIs), a broader term covering the use of digital technologies to support and improve health and health care [5-7], include remote patient monitoring (RPM). RPM enables health care providers to monitor patients’ health status remotely, facilitating timely interventions and potentially improving health outcomes. It has gained attention as a strategy to optimize resource use and enhance chronic care management worldwide [7]. In Norway, a national implementation program for RPM was launched in 2022 to address these challenges [8]. Assessing its economic impact is essential for informing policy decisions and ensuring sustainable implementation. This review provides a foundation for such evaluation by exploring existing economic evaluation approaches used in RPM research. The findings will contribute to the ongoing assessment of the Norwegian program and support future evidence generation on its cost-effectiveness.

The literature on RPM encompasses a wide array of technologies, services, and delivery approaches. This body of research is characterized by significant fragmentation, as it spans a diverse range of chronic conditions, heterogeneous health care settings, and multiple levels of care [1,5,7]. Consequently, the economic evidence of RPM exhibits variability across the spectrum of chronic conditions and within different health care contexts [9-12].

In an era of rising health care costs and limited resources, economic evaluations have become indispensable tools for informed decision-making in health care systems worldwide [13,14]. These evaluations provide a systematic framework for comparing the costs and consequences of different health care interventions, enabling policy makers, health care providers, and payers to allocate resources more efficiently and effectively [15,16]. Economic evaluations play a role in ensuring the sustainability of health care systems and maximizing population health outcomes by bridging the gap between clinical efficacy and real-world cost-effectiveness [13,17].

Health care interventions, including digital health solutions, require a rigorous economic assessment to justify their implementation and continued use [18,19]. Several well-established methodologies are used in health economic evaluations, each with its specific focus and application [14,20,21]:

- Cost-effectiveness analysis (CEA) evaluates the relative costs and health outcomes of alternative interventions, typically reporting results as cost per unit of health outcome gained.
- Cost-utility analysis (CUA) a subtype of CEA, uses quality-adjusted life years (QALYs) to capture both the quantity and quality of life, facilitating broader comparisons across health care interventions.
- Cost-minimization analysis (CMA) applies when outcomes are equivalent across alternatives, focusing solely on identifying the least costly option.
- Cost-benefit analysis quantifies both the costs and benefits of an intervention in monetary terms, enabling the assessment of net benefit to society.
- Cost-consequence analysis (CCA) lists costs and outcomes separately, without aggregating them, allowing decision-makers to weigh multiple dimensions transparently.

In addition, cost analysis (CA) and budget impact analysis (BIA) provide complementary insights [22,23]. CA examines direct and indirect costs, without linking them to outcomes, while BIA estimates the financial implications of adopting a new intervention within a specific health care budget context.

Economic evaluation of DHIs and RPM poses unique challenges compared to traditional interventions. Their complex, multifaceted components make it difficult to isolate specific intervention effects [10,11,24,25]. Recent literature advocates for standardized evaluation approaches [24,26-29], as current methods inadequately capture the full spectrum of outcomes, particularly nonhealth and process-related impacts. Several reviews propose developing tailored frameworks and methodologies to address these evaluation gaps.

Recent reviews of DHIs have explored methods for program cost [30] and assessment of clinicians’ time [4]. However, previous reviews in this field have overlooked the details of costing methods used in economic evaluations. This gap underscores the need for an examination of the practical approaches to cost estimation, including cost identification, measurement, and valuation. To address this knowledge gap, our scoping review aims to provide a comprehensive examination of how costs are handled in current studies on DHIs, particularly in the context of RPM.

This scoping review aims to explore the economic evaluation methodologies applied to RPM for chronic conditions, with a particular focus on how costs are identified, measured, and valued. By synthesizing current research, the review seeks to highlight existing methodological approaches, assess reporting quality, and identify gaps to inform future research and policy.

## Methods

### Study Design

A scoping review was chosen for this study to systematically chart and explore the existing evidence on economic evaluations within the context of RPM for chronic conditions. This review follows the Joanna Briggs Institute methodology for scoping reviews, which aligns with the PRISMA-ScR (Preferred Reporting Items for Systematic Reviews and Meta-Analyses Extension for Scoping Reviews) guidelines [31]. The detailed protocol for this review has been registered in the Open Science Framework [32]. To investigate the quality of the reporting in the economic evaluation studies, we adhered to the CHEERS (Consolidated Health Economic Evaluation Reporting Standards) checklist [33].

### Eligibility Criteria

The inclusion criteria include (1) studies targeting patients with CVD, CRD, DM, psychiatric disorders, and cancer as the focus of RPM; (2) studies conducting economic evaluation of RPM interventions, including CEA, CUA, CMA, CCA, CA, and BIA; (3) studies conducted in various health care settings, encompassing different levels of care, such as primary care, specialist care, and other relevant health care contexts. No geographical restrictions, beyond requiring English-language publications; and (4) empirical studies using randomized controlled trial design, pre-post studies, or observational designs.

The exclusion criteria include (1) studies not directly related to chronic conditions or RPM; (2) studies not involving economic evaluations, BIA, or cost aspects in the context of RPM for chronic conditions; (3) studies conducted exclusively in nonhealth care settings or unrelated to care delivery; and (4) exclude editorials, commentaries, opinion pieces, and studies lacking sufficient detail or relevance to the research question. We excluded modeling studies, even if they used empirical inputs, to focus directly on observed data.

This approach allows for a richer exploration of cost identification, measurement, and valuation in real-world RPM settings.

### Information Sources and Search Strategy

A research librarian assisted in developing and executing the search strategy across four electronic databases: Embase (Ovid), MEDLINE (Ovid), Web of Science (Core Collection), and CINAHL (Ebsco) in week 40 of 2023. The strategy combined relevant subject headings (eg, MeSH and Emtree terms) and free-text terms related to digital health (eg, telemedicine, telehealth, remote care, eHealth, mHealth), chronic conditions (eg, multimorbidity, CVD, and diabetes), and economic evaluations (eg, cost-effectiveness, cost-utility, and health economics). Reviews were screened for additional relevant references not identified through database search. The full search strategy for each database is provided in Multimedia Appendix 1.

### Study Selection

The bibliographic references were imported into Rayyan, a web-based tool designed to facilitate the selection of studies for systematic review screening. The Rayyan tool is also useful for other literature review methods, like this scoping review. Before beginning the full screening process, the review team conducted a calibration exercise on a sample of 20 papers to ensure consistent interpretation of the inclusion and exclusion criteria. Titles and abstracts screening was then independently performed by two independent reviewers (SB and EB), applying the predefined eligibility criteria. The papers were included if they met the eligibility criteria. After the initial screening, selected papers underwent a full-text review, which was also conducted independently by both reviewers (SB and EB). Any discrepancies at either stage were resolved through discussion until a consensus was reached.

### Data Extraction and Management

The data extraction process involved designing and creating an Excel (Microsoft Corp) form for consistency across the reviewers. The form included fields for title, authors, publication year, country, study design, methods, diagnosis, comparator, cost estimation details, outcome measures, intervention descriptions, and main findings. Extracting data was categorized according to four key themes: study characteristics (design, population, and setting), economic evaluation methods (types of analysis, comparator, perspectives, and outcome measures), cost estimation (identification, measurement, and valuation), and adherence to CHEERS. This thematic structure facilitated synthesis and comparison across studies.

Study quality was assessed using the CHEERS guidelines and checklist by SB and EB independently, with disagreements resolved by discussion.

### Data Synthesis

Data synthesis was conducted using a thematic approach, systematically organizing the collected data. This process focused on categorizing and analyzing the findings of the included studies, identifying common themes or patterns across them. The synthesis comprehensively explored the various cost identification, measurement, and valuation methods used in economic evaluations of RPM for chronic conditions.

## Results

### Sample

**Figure 1.**
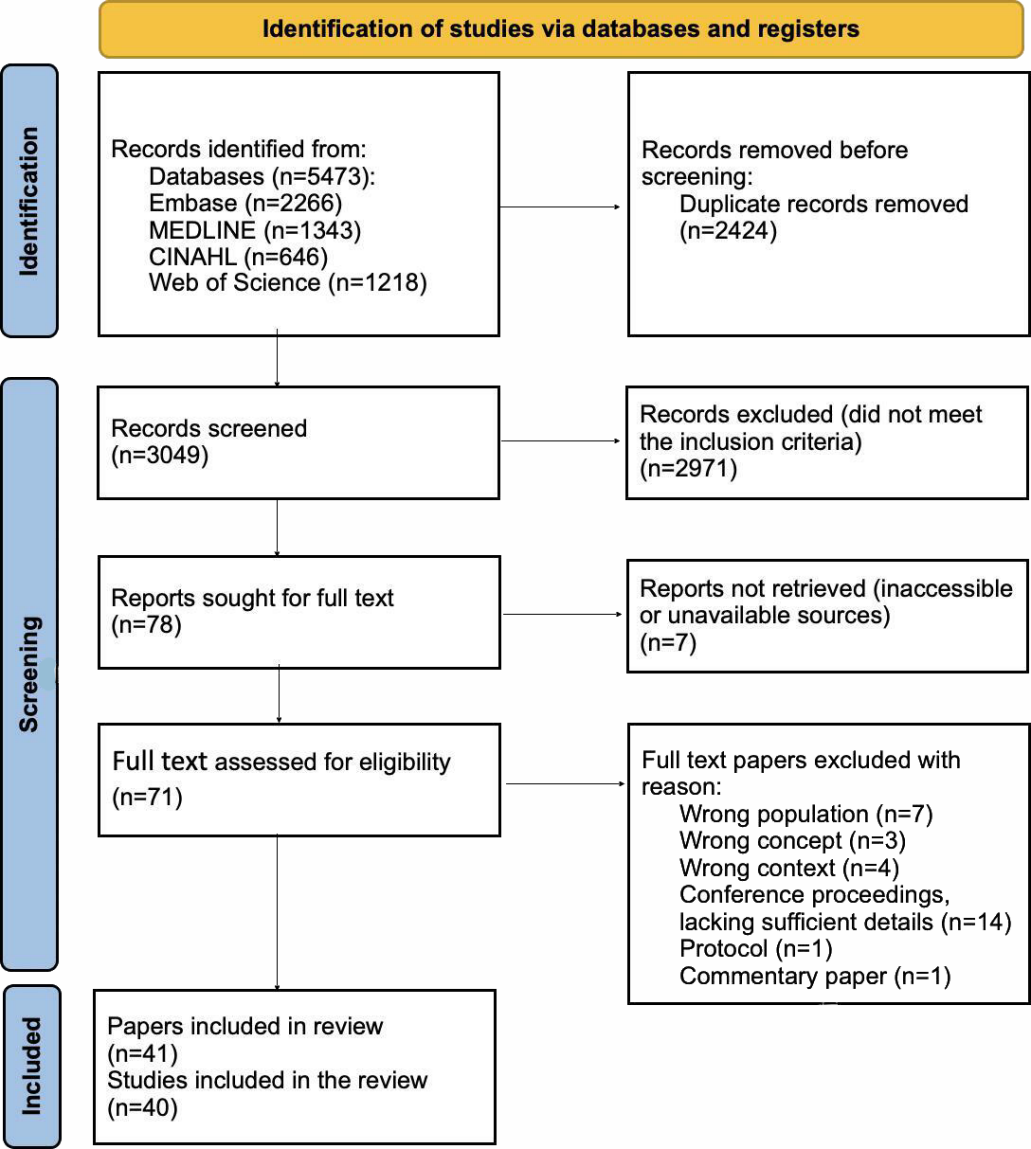
Flowchart of the search and screening of papers.

A total of 3049 records were identified in the search. Two of the papers refer to the same study but one as a subgroup analysis, so both were retained. The screening began with a title and abstract review, which excluded 2971 records not meeting the inclusion criteria, leaving 78 papers for full-text assessment. Seven records were inaccessible or unavailable and could not be retrieved, resulting in 71 full texts assessed against the inclusion criteria. Based on the full-text assessment, an additional 30 were excluded for various reasons (Figure 1). After exclusions, 41 papers, representing 40 studies, were included in the final review.

### Study Characteristics

The included studies reveal a diverse range of medical conditions, perspectives, study designs, types of analysis, and outcome measures, summarized in Table 1. All the studies comparator, besides the BIA, are usual care.

**Table 1.** Study characteristics and economic evaluation details of included studies.

Author (year), country	Main diagnosis	Study perspective	Study design	Type of analysis	Outcome measure
Achelrod et al (2017), Germany [34,35][Bibr R34]	CRD^a^ (COPD)^b^	Payer	Matched control	CEA^c^	Mortality
Apantaku et al (2022), Canada [36]	CVD^d^	Partial societal	Pre-post	CCA^e^	Cost savings
Blum and Gottlieb (2014), United States [37]	CVD	Payer	RCT^f^	CEA	Costs, 30-day readmission rates, mortality
Carter et al (2023), Australia [38]	CVD, CRD, DM^g^, or CKD^h^	Provider	Pre-post	CCA	Health care resource use, costs, and patient-reported outcomes
Chen et al (2013), Taiwan [39]	CVD	Provider	Pre-post	CEA	Costs
Clarke et al (2018), United Kingdom [40]	CRD (COPD)	Provider	Pre-post	CEA	Resource use
Comin-Colet et al (2016), Spain [41]	CVD	Provider	RCT	CCA	Hospital costs, nonfatal HF^i^ events
de Batlle et al (2021), Spain [42]	Complex chronic conditions	Provider	Matched control	CEA	SF-12^j^, health care resources
Deng et al (2015), Canada [43]	DM	Patient	Matched control	CBA^k^	Costs associated with doctor appointments
Esteban et al (2021), Spain [44]	CRD (COPD)	Provider	Matched control	CUA^l^	QALY^m^
Finkelstein et al (2006), United States [45]	CVD, CRD (COPD), or chronic wound	Provider	RCT	CCA	Costs, mortality, morbidity
Frederix et al (2019), Belgium [46]	CVD	Health care sector	RCT	CCA	All-cause mortality, health care costs, and heart failure admissions
Greving et al (2015), Netherlands [47]	CVD	Societal	RCT	CUA	QALY
Henderson et al (2013), United Kingdom [48]	CVD, CRD (COPD), or DM	Health care sector	RCT	CUA	QALY
Herold et al (2018), Germany [49]	CVD	Payer	Matched control	CA^n^	Total health care costs
Ho et al (2014), Taiwan [50]	CVD	Health care sector	Matched control	CEA	Hospitalization, ED^o^ visits
Inocencio et al (2023), United States [51]	CRD (COPD)	Payer		BIA^p^	3-year budget impact
Isaranuwatchai et al (2018), Canada [52]	CRD (COPD) or CVD	Provider	Pre-post	CA	Number and costs of hospitalizations and ER visits
Jódar-Sánchez et al (2014), Spain [53]	CRD (COPD)	Provider	RCT	CUA	QALY
Lee et al (2021), Taiwan [54]	CVD	Provider	Matched control	CEA	Clinical end points, medical costs
Maeng et al (2014), United States [55]	CVD	Payer	Pre-post	CCA	RoI^q^, cost savings, probability of readmission
Rubio et al (2018), Spain [56]	CRD (COPD)	Provider	Pre-post	CA	Incidence and mean duration of hospital admissions and incidence of ER visits
Mudiyanselage et al (2019), Australia [57]	CRD (COPD) or DM	Provider	RCT	CUA	QALY
Mudiyanselage et al (2023), Australia [58]	CRD (COPD) or DM	Provider	RCT	CUA	QALY
Noel et al (2004), United States [59]	CVD, CRD (COPD), or DM	Provider	RCT	CCA	Health resource use, costs, QoL^r^
Palmas et al (2010), United States [60]	DM	Payer	RCT	CA	Health care utilization
Paré et al (2006), Canada [61]	CRD (COPD)	Provider	Matched control	CMA^s^	Costs
Paré et al (2013), Canada [62]	CRD (COPD)	Provider	RCT	CMA	Costs
Paré et al (2013), Canada [63]	CVD, CRD (COPD), DM, or hypertension	Provider	Pre-post	CMA	Costs
Pathak et al (2022), France [64]	CVD	Payer	Matched control	CA	Total costs of health care consumption
Riley et al (2015), United States [65]	CVD	Payer	Matched control	CA	Health care utilization
Sohn et al (2012), Germany [66]	CVD	Payer	Matched control	CBA	Health care costs, mortality, HRQoL^t^
Stoddart et al (2015), United Kingdom [67]	CRD (COPD)	Provider	RCT	CUA	QALY
Sydow et al (2022), Germany [68]	CVD	Payer	RCT	CUA	Costs per day alive and out of hospital, and cost per QALY
Vestergaard et al (2020), Denmark [69]	CVD	Health care sector	RCT	CUA	QALY
Warren et al (2018), Australia [70]	DM	Provider	RCT	CUA	HbA_1c_
Willems et al (2007), Netherlands [71]	CRD (Asthma)	Health care sector	RCT	CUA	QALY
Udsen et al (2017), Denmark [72]	CRD (COPD)	Health care sector	RCT	CUA	QALY
Udsen et al (2017), Denmark [73]	CRD (COPD)	Health care sector	RCT	CUA	QALY
Zaman et al (2023), United Kingdom [74]	CVD	Provider	Matched control	CA	Secondary health care use and costs
Ziegler et al (2023), Germany [75]	CVD	Payer	RCT	CUA	QALY

a CRD: chronic respiratory disease.

b COPD: chronic obstructive pulmonary disease.

c CEA: cost-effectiveness analysis.

d CVD: cardiovascular disease.

e CCA: cost-consequence analysis.

f RCT: randomized controlled trial.

g DM: diabetes mellitus.

h CKD: chronic kidney disease.

i HF: heart failure.

j SF-12: 12-Item Short Form Health Survey.

k CBA: cost-benefit analysis.

l CUA: cost-utility analysis.

m QALY: quality-adjusted life year.

n CA: cost analysis.

o ED: emergency department.

p BIA: budget impact analysis.

q RoI: return on investment.

r QoL: quality of life.

s CMA: cost-minimization analysis.

t HRQoL: health-related quality of life.

The distribution of chronic conditions examined in the included studies shows that CVD was the most common focus in single-diagnosis studies, with 17 studies [36,37,39,41,46,47,49,50,54,55,64-66,68,69,74,75], followed by CRD, represented in 12 studies [35,40,44,51,53,56,61,62,67,71-73]. Within CRD, chronic obstructive pulmonary disease (COPD) was specifically addressed in 11 of these studies, and asthma in 1 study [71]. DM was the focus of 3 studies [43,60,70]. Additionally, 9 studies involved multiple chronic conditions, mostly CVD, COPD, or DM in the same study [38,42,45,48,52,57-59,63]. These studies span a broad range of countries, with the majority originating from high-income countries in Europe, North America, and Australia.

### Study Design and Method

In this review, a variety of study designs and types of economic analyses are used across the included studies. Randomized controlled trials emerged as the predominant study design, accounting for 19 of the total studies included [41,45-48,53,57-60,62-73,75]. Moreover, matched control group designs were used in 12 studies [34,42-44,49,50,54,61,64-66,74], while 8 studies used a pre-post single-group design [36,38-40,52,55,56,63].

In terms of economic analyses, we classified studies based on the method of economic evaluation reported by the authors themselves, with adjustments made when methods were not explicitly reported. CEA emerged as the predominant approach, with 20 studies using this method [34,37,39,40,42,44,47,48,50,53,54,57,58,67-73,75]. Within CEA, 13 studies specifically conducted CUA [44,47,48,53,57,58,67-73,75]. The remaining studies showed diverse methodological approaches: 7 studies conducted CCA [36,38,41,45,46,55,59], 2 studies carried out cost-benefit analysis [43,66] and 3 studies conducted CMA [61-63], aimed at identifying interventions with equivalent effectiveness but differing costs. A total of 8 studies used CA methods [49,50,52,56,60,64,65,74], with 1 study focused exclusively on BIA [51].

### Outcome Measures

Various economic outcome measures were reported across the studies, including mortality, QALYs, hospital readmission rates, and cost reductions. QALYs were a prominent measure in studies using CUA, with interventions for conditions like COPD and heart failure showing mixed results in cost-effectiveness. For instance, Stoddart et al [67] found a 15% probability of being cost-effective for COPD at £30,000 (US $40,685.41) per QALY, while Sydow et al [68] found heart failure interventions to be cost-effective when measured by QALY improvements. Other outcome measures included health care costs (eg, Herold et al [49] and Riley et al [65].

### The Estimation of Costs

The second key aspect outlined in this scoping review is to describe cost identification, measurement, and valuation methods summarized in Table 2. More details about the cost categories can be found in Multimedia Appendix 2 [34-75].

**Table 2.** Cost categories, cost measurements, and cost valuation.

Author (year)	Cost categories	Cost measurement	Cost valuation
Achelrod et al (2017) [34,35]	Health care utilization, pharmaceuticals, and rehabilitation costs	Claims data (AOK Bayern, public health insurer)	Reimbursement rates
Apantaku et al (2022) [36]	Health care utilization, out-of-pocket costs	Patients’ self-reported health care use	Published national unit costs by Canadian Ministry of Health and Canadian Institute of Health Information
Blum and Gottlieb (2014) [37]	Number and days of hospitalization, and emergency department (ED) visits costs	Claims data (health insurer)	Reimbursement rates
Carter et al (2023) [38]	Health care utilization	Administrative databases for the MeCare program	Total costs of the MeCare Program
Chen et al (2013) [39]	Health care utilization	Hospital claims data	Reimbursement rates
Clarke et al (2018) [40]	Health care utilization and program costs	Administrative databases and hospital claims data	National Health Service Direct reimbursement rates and Primary Care Trusts rates
Comin-Colet et al (2016) [41]	Health care utilization	Electronic medical records and interviews of health care personnel	Hospital reimbursement rates
de Batlle et al (2021) [42]	Health care utilization	Electronic medical record	Catalan Institute of Health official pricing or reimbursement rates
Deng et al (2015) [43]	Patient travel and time use	Patient self-reported health care use	Patient-reported costs
Esteban et al (2021) [44]	Health care utilization	Claim data	Reimbursement rates
Finkelstein et al (2006) [45]	Digital visits	Acquired equipment	Mileage reimbursement, staff compensation, overhead, market prices
Frederix et al (2019) [46]	Health care utilization	Claim data	Reimbursement rates
Greving et al (2015) [47]	Health care utilization, intervention, and patient travel	Patient self-reported health care use, electronic patient journal, and time assessment of health care personnel	Dutch Healthcare Costing and Drug Information Guidelines, medication price or reimbursement rates
Henderson et al (2013) [48]	Telehealth equipment, telehealth support, health care utilization, and social care use	Patient-reported service use and information from each site	Market prices, personnel costs, and national unit costs or reimbursement rates
Herold et al (2018) [49]	Health care utilization	Hospital claims data	Reimbursement rates
Ho et al (2014) [50]	Health care utilization, patients’ self-payment, and intervention	Electronic database at the hospital	Reimbursement rates
Inocencio et al (2023) [51]	Health care utilization	Peer-reviewed literature, direct evidence of use of the service	Peer-reviewed literature adjusted to a Medicare payer population
Isaranuwatchai et al (2018) [52]	Health care utilization and program costs	Alaya care mobile app	Canadian Institute of Health Informatics unit cost or reimbursement rates
Jódar-Sánchez et al (2014) [53]	Health care utilization, professionals’ intervention, telehealth system	The service supplying company and time estimations	Andalusian Health Service reimbursement rates and the service supplying company
Lee et al (2021) [54]	Health care utilization	Medical records	Reimbursement rates
Maeng et al (2014) [55]	Health care utilization	Geisinger Health Plan claims data (health insurer)	Geisinger Health Plan reimbursement rates
Rubio et al (2018) [56]	Health care utilization	Nurse scheduled working hours and electronic health records	Unit cost publication by the Galician Health Service
Mudiyanselage et al (2019) [57]	Health care utilization and intervention costs	Hospital admission system	Hospital costing system
Mudiyanselage et al (2023) [58]	Health care utilization, intervention costs	Study team, service data, and patients’ self-report	DRG^a^, Hospital LOS^b^ information, Victorian Department of Health’s Weighted Inlier Equivalent Separation calculator, unit prices
Noel et al (2004) [59]	Health care utilization	Health provider’s electronic database and home-based program, community agencies, and distances	Health provider’s claims data, and transportation costs
Palmas et al (2010) [60]	Health care utilization and project intervention costs	Medicare claims (health insurer), vendors’ individual contracts, and project costs	Medicare reimbursement, vendors’ individual contracts, and actual expenditures
Paré et al (2006) [61]	Health care utilization, and technology costs	Management control systems, and patient medical records	Mean hourly rats, DRGs, and market prices
Paré et al (2013) [62]	Health care utilization and technology costs	Number and length of home visits and negotiated prices	Average hourly rate, mileage rate, and negotiated prices
Paré et al (2013) [63]	Health care utilization, intervention costs, and technology costs	Computerized medical records, information systems at the JR Health Center, scheduled time use, and negotiated prices	Average hourly rate, mileage rate, and negotiated prices
Pathak et al (2022) [64]	Health care utilization	French National Health Insurance Data System	Official French national tariffs 2019 or reimbursement rates
Riley et al (2015) [65]	Health care utilization	Hospital charges	Hospital charges
Sohn et al (2012) [66]	Health care utilization	The health insurance database	The health insurance reimbursement rates
Stoddart et al (2015) [67]	Health care utilization and intervention costs	Anecdotal descriptions from staff, contracted services, time sheets, questionnaires, previous surveys, recordings, and patients’ secondary care records	Average hourly wage, standard UK price weights, British National Formulary, and weighted averages in nonrespiratory wards
Sydow et al (2022) [68]	Health care utilization and intervention costs	Statutory health insurance claims data	Statutory health insurance claims data
Vestergaard et al (2020) [69]	Health care utilization and intervention costs	Registers, registrations, licenses, and payments to supplier	DRGs^a^, GP^c^ fees, market prices, wages, estimated prices (nursing homes), licenses, payments, and expected purchase price
Warren et al (2018) [70]	Health care utilization and intervention costs	Data collected by the research team, information from GPs, and public hospitals	DRGs and actual trial costs
Willems et al (2007) [71]	Health care utilization, intervention, patient and family costs, and productivity losses	Hospital billing system, patient cost diary, time registration, and data collected by the research team	Dutch manual for cost research, Ministry of Education, Culture, and Science, unit prices, salary
Udsen et al (2017) [72]	Health care utilization and intervention costs	Danish registers, individual care systems in municipality districts, planned time for workshops	DRGs, reference prices, negotiated fees, consumer price, average, prices paid, and negotiated prices
Udsen et al (2017) [73]	Refer to Udsen et al, 2017 [72]	Refer to Udsen et al, 2017 [72]	Refer to Udsen et al, 2017 [72]
Zaman et al (2023) [74]	Secondary health care, and platform costs	Electronic health record and the Discover data platform	Details not reported
Ziegler et al (2023) [75]	Health care utilization and intervention costs	Individual cost data	Health insurance claims data, actual labor costs, prices, and assumed infrastructure costs

a DRG: diagnosis-related group.

b LOS: length of stay.

c GP: general practitioner.

The selection of cost components to include in each study depended on the specific perspective adopted and the focus of the economic evaluation conducted. The included studies considered a broad range of cost components. In the Danish studies, the perspective was that of the public payer, specifically the Danish public health care system, which included costs for prehospital services, in-patient and out-patient care, primary health care, prescription medicines, and intervention costs [69,72,72]. A total of 2 studies adopted a societal perspective, incorporating patient and family costs, productivity losses, and health care utilization and intervention costs [47,71]. Studies focusing on health insurance companies included costs relevant to the insurance plan, such as hospital services, ambulatory care, prescription medicines, and medical devices, while excluding costs like telemonitoring if not directly borne by the insurer [34,37,51,66,75]. German statutory health insurance studies, costs associated with hospital services, prescription medicines, and other covered interventions were included [34,49,66,68]. Studies from the perspective of hospitals or health services have primarily included costs related to hospital services, medical treatments, consultations, outpatient care, and interventions provided by the respective institutions [36,38,40,41,44,45,48,50,52,53,57,58,60,62,64,74]. Studies involving home health care agencies included costs associated with providing home-based health care services, such as home visits, medical treatments, and other related expenses [40,61,62,67]. In total, 10 of the studies did not report inclusion of the intervention costs [39,42,43,46,49,52,54,56,59,65].

Our synthesis identified variations in the data sources used for cost estimation and how they were used to derive cost values. We also observed a diverse array of terminology used in the publications to denote comparable sources. For example, many studies relied on electronic health records, patient administration systems, and hospital electronic databases as sources for cost measurement. Some studies reported health care utilization based on patient-recorded visits (eg, Apantaku et al [36] and Henderson et al [48]), while another study combined patient-recorded data with electronic patient records [47].

For cost valuation and unit cost estimation, studies used diverse approaches, including tariffs and official price lists. Diagnosis-related group tariffs were commonly used [58,61,69,72,73]. National or regional costing guidelines were applied, such as the Dutch manual for cost research [71], Official French national tariffs 2019 [64], and the Catalan Institute of Health official pricing [42]. Health insurance claims served as a cost source in multiple studies [37,49,52,60,68,75] and standard price weights were used in the UK context [67]. One study was identified as a BIA, using unit cost data and medication use from published studies and adjusted to align with the payer of the study population [51].

In addition to the diverse methods of costing observed, the reviewed studies applied both microcosting and gross-costing methodologies. Some studies provided detailed cost breakdowns, while others relied on aggregated estimates.

### Study Quality Reporting Assessment

**Figure 2.**
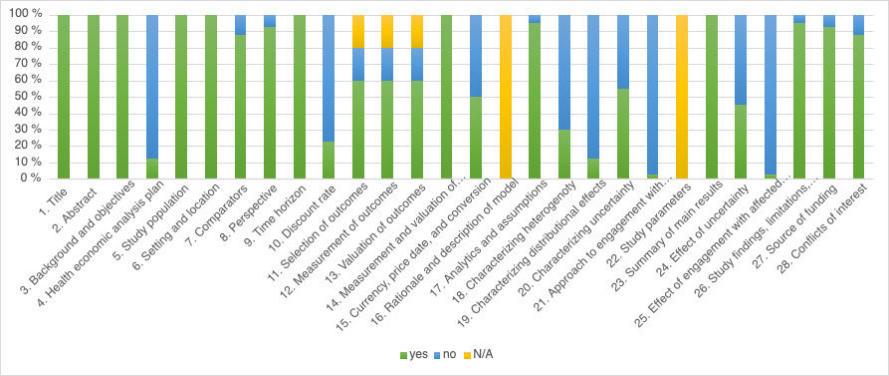
Bar graph for CHEERS percentage score for each checklist item. CHEERS: Consolidated Health Economic Evaluation Reporting Standards; N/A: not applicable.

The studies varied in terms of intervention types, populations, and settings, but all were assessed based on their adherence to the CHEERS checklist items, as illustrated in Figure 2. The BIA study was not evaluated against the CHEERS checklist, as it is not applicable to this type of analysis. For more details, see the CHEERS checklist items (Checklist 1) and the study-specific CHEERS 2022 adherence table available in Multimedia Appendix 3.

Assessment of reporting quality using the CHEERS checklist revealed varying levels of adherence across different criteria. All studies (40/40, 100%) adequately described their study population and inclusion or exclusion criteria. Most studies (35/40, 88%) clearly identified relevant comparators and justified their chosen time horizons (100%). While all studies, except the BIA study, had usual care as a comparator, the CHEERS checklist applies a stricter definition of a comparator, which may account for differences in reporting.

However, several key reporting elements showed notable gaps. Only 23% (9/40) of papers reported their discount rate, while 60% (24/40) addressed the selection, measurement, and valuation of health outcomes. Critical methodological elements were often underreported; 50% (20/40) of studies detailed the dates of estimated resource quantities and unit costs, including currency and conversion information. Similarly, 55% (22/40) described methods to characterize uncertainty in their analyses. Less than half (18/40, 45%) of the studies reported how uncertainty about analytic judgments, inputs, or projections affected their findings, including choices regarding discount rates and time horizons.

The newer elements introduced in the CHEERS 2022 checklist received minimal attention across the reviewed studies. Few papers addressed items such as health economic analysis plans, characterization of heterogeneity and distributional effects, or stakeholder engagement during study design and implementation. This limited reporting likely reflects the timing of these studies, many of which were conducted before or shortly after the updated guidelines’ release.

## Discussion

### Principal Findings

This review highlights the variability in economic evaluation methodologies used in RPM studies and the inconsistent application of cost identification, measurement, and valuation methods. The included studies applied different approaches to defining and measuring costs, contributing to challenges in comparability.

Reported economic outcomes for RPM varied, with some studies identifying cost savings and improvements in health-related measures. This variation aligns with findings in previous reviews on DHIs [9-12,25,27,76-78].

Traditional health economic evaluation methods primarily focus on measuring health outcomes, often through metrics like QALYs, which may not fully capture the nonhealth and process outcomes relevant to DHIs, including RPM [24,26-28]. For instance, equity impacts, patient satisfaction, and process improvements are crucial, but often overlooked. Gomes et al [24]and Benedetto et al [26] have highlighted these limitations and proposed alternative approaches, such as impact matrices and CCA, which offer a broader view of the outcomes. The National Institute for Health and Care Excellence (NICE) similarly recommends considering both process and health outcomes in economic evaluations, pointing to examples such as reduced outpatient consultations as relevant process improvements [79].

A key challenge identified in the review was the inconsistent application of resource costing methods. The three main components, cost identification, measurement, and valuation, were often reported with limited transparency. This might bias results, make cross-study comparison difficult, and thereby, limit generalizability. In the field of health economics, accurately estimating resource costs is crucial for conducting robust CA and cost-effectiveness studies [14,23]. Resource costing methods are the primary costing approach for assessing the economic implications of health care interventions, diseases, and utilization patterns. The specific context of the economic evaluation further influences how resource quantities are determined. Cost identification involves assessing all relevant resources consumed, from direct medical costs to indirect costs like patient travel and productivity losses [14]. However, study perspectives varied, influencing which costs were considered.

Cost measurement approaches also differed, with some studies using the microcosting approach, a detailed assessment, and others relying on macrocosting, using aggregated cost data. The choice between these approaches depends on data availability and study objectives, though inconsistent application adds variability. The valuation step assigns unit prices to each resource, ideally reflecting opportunity cost, though practical factors often lead to using prevailing market prices and tariffs, further complicating consistency. Factors like regional price differences, choice of price weights, and whether charges or actual costs are used can affect generalizability and comparability across studies [14].

Despite these established principles, the practical application of resource costing in our review remains inconsistent. Many studies lacked transparency in cost identification, measurement, and valuation details, making comparisons difficult. Challenges also include adjustments for time (eg, discount rates) and inflation, as well as the transparency of data sources. Moreover, the absence of detailed tracking systems, particularly in settings with block funding, limits the precision of cost analyses. In such cases, reliance on broad estimates instead of granular data can affect the accuracy of cost evaluations and bias the results [14]. Additionally, task-shifting, delegating certain responsibilities to less specialized health workers, can influence cost outcomes but was underexplored in the reviewed studies.

The limited use of BIA across studies, seen in only one, points to gaps in assessing affordability alongside cost-effectiveness. While the International Society for Pharmacoeconomics and Outcomes Research acknowledges BIA as essential for a comprehensive economic evaluation [80,81], few studies implemented it, leaving a need for more comprehensive financial assessments.

Another limitation of the current evidence base is its exclusive focus on high-income countries. The absence of studies from low- and middle-income countries creates a knowledge gap, particularly given that these regions might benefit from remote monitoring solutions to address health care access challenges.

The updated CHEERS 2022 standards call for thorough reporting in economic evaluations, including cost-analysis plans and subgroup analyses. However, most of the studies in this review predated or only partly conformed to these updated guidelines. The inconsistent application of sensitivity analyses, time discounting, inflation adjustments, and discount rates further complicated comparisons across studies. Standardizing these practices would improve both transparency and comparability.

Finally, the practical aspects of conducting cost analyses in DHIs also require attention. For instance, accurately measuring health care professionals’ time spent on RPM activities can be challenging in the absence of standardized unit prices. Additionally, studies often failed to adjust for inflation or to use appropriate discount rates when presenting cost data, making comparisons between studies difficult. Future studies should aim for more transparent and detailed reporting on these practical aspects to enhance the reliability and generalizability of their findings.

### Strengths and Limitations

This review’s strengths include its comprehensive search strategy, systematic approach to data extraction, and detailed quality assessment using the CHEERS checklist. However, several limitations should be noted. First, the focus on specific chronic conditions aligned with Norway’s implementation program may have excluded relevant studies in other conditions. Second, the heterogeneity in reporting styles and methodologies made direct comparisons challenging. Another limitation is the search date of this review, week 40 of 2023, leaving possible relevant studies published in 2024 out. Finally, the rapid evolution of digital health technologies means some newer implementation models may be underrepresented in the literature.

### Recommendations for Future Research and Policy

Future research should prioritize (1) the development of standardized reporting frameworks specifically adapted for DHIs, (2) investigation of RPM implementation in low- and middle-income countries, (3) integration of broader outcome measures beyond traditional health economic metrics, (4) increased use of budget impact analyses alongside cost-effectiveness studies, and (5) examination of long-term economic impacts and sustainability of RPM programs.

Policy makers play a crucial role in fostering the advancement and adoption of evidence-based RPM strategies and they should actively support research initiatives addressing the identified knowledge gap, particularly in areas such as standardized reporting, BIA, and implementation in diverse health care settings. Established funding mechanisms for rigorous economic evaluation will also incentivize researchers to conduct high-quality studies that inform decision-making. Furthermore, initiatives should be put in place to foster collaboration among researchers, health care providers, and relevant stakeholders to facilitate the translation of research findings into real-world practice.

### Conclusions

This review underscores the variability in economic evaluations of RPM and the inconsistent reporting of cost identification, measurement, and valuation. The limited use of BIA and the lack of studies from low- and middle-income countries highlight gaps in the literature.

There is an increasing acknowledgment of the need for more comprehensive methodological approaches, including the integration of process outcomes and BIA. Furthermore, adherence to updated reporting standards like CHEERS 2022 is still limited, with most studies not including key elements like sensitivity analyses or detailed cost estimation practices.

Inconsistent application of time discounting, inflation adjustments, and resource costing principles, along with a lack of transparency in cost data, further complicates the interpretation of findings. Establishing standardized, transparent reporting protocols will advance this field and significantly enhance the comparability and generalizability of economic impact assessments for DHIs.

## Supplementary material

10.2196/71565 Multimedia Appendix 1 Search strategy.

10.2196/71565 Multimedia Appendix 2 Overview of cost categories, cost measurement, and cost valuation of included studies.

10.2196/71565 Multimedia Appendix 3 Study-specific CHEERS (Consolidated Health Economic Evaluation Reporting Standards) 2022 adherence table.

10.2196/71565 Checklist 1 CHEERS (Consolidated Health Economic Evaluation Reporting Standards) 2022 checklist.

10.2196/71565 Checklist 2 PRISMA-ScR (Preferred Reporting Items for Systematic Reviews and Meta-Analyses Extension for Scoping Reviews) checklist.

## Abbreviations

BIA: budget impact analysis
CA: cost analysis
CCA: cost-consequence analysis
CEA: cost-effectiveness analysis
CHEERS: Consolidated Health Economic Evaluation Reporting Standards
CMA: cost-minimization analysis
COPD: chronic obstructive pulmonary disease
CRD: chronic respiratory disease
CUA: cost-utility analysis
CVD: cardiovascular disease
DHI: digital health intervention
DM: diabetes mellitus
PRISMA-ScR: Preferred Reporting Items for Systematic Reviews and Meta-Analyses Extension for Scoping Reviews
QALY: quality-adjusted life year
RPM: remote patient monitoring
