# Supplementary material for: Economic Evaluation Methodologies of Remote Patient Monitoring for Chronic Conditions: Scoping Review
Source: J Med Internet Res. 2025 Jul 4;27:e71565. doi: 10.2196/71565 (PMC12248258; doi:10.2196/71565)
Supplement: Multimedia Appendix 1 [file jmir-v27-e71565-s001.docx]

**Embase <1974 to 2023 October 02> via OVID**

1 *Telemedicine/ or *Telenursing/ or Mobile Applications/ or Smartphone/ or (telemedicine or tele-medicine* or telehealth or tele-health or telecare or tele-care or telehealthcare or tele-healthcare or telehome* or tele-home* or telemonitoring or tele-monitoring or telenurs* or tele-nurs* or telesupport* or tele-support* or telemental* or tele-mental* or telesurveillance or tele-surveillance or telefollow* or tele-follow* or telepatient* or tele-patient* or telemanag* or tele-manag* or e-care or ecare or e-health or ehealth or m-health or mhealth or mobile health or m-care or mcare or e-mental* or emental* or m-mental or mmental or telepsychiatry or tele-psychiatry or ((home or remote or digital) adj3 monitoring) or ((remote or distant or distance* or tele*) adj3 (care or healthcare or patient*)) or ((mobile or smartphone or tablet) adj3 (app or apps or application*)) or (iphone* or ipad* or smart device* or smartphone* or smart-phone* or web-based or online)).ti,ab,kw. 507819

2 Multiple Chronic Conditions/ or Multimorbidity/ or Comorbidity/ or Chronic disease/ or (multimorbid* or complex-care or multiple-condition* or multiple-chronic-condition* or chronically ill or ((long-term or chronic or co-morbid) adj (condition* or disease* or diagnosis* or illness*)) or (chronic adj (comorbidit* or co-morbidit*))).ti,ab,kw. 712762

3 exp Cardiovascular Diseases/ or (cardio* or cardia* or heart* or coronary* or angina* or ventric* or myocard* or pericard* or isch?m* or emboli* or arrhythmi* or thrombo* or atrial fibrillat* or tachycardi* or endocardi* or sick sinus or hypertension* or stroke or ((vascular or cerebrovascular or arterial) adj (disease* or disorder*)) or ventricular).ti,ab,kw. 6457284

4 Noncommunicable Diseases/ or ((non-communicable or non-infectious or noncommunicable or noninfectious or chronic) adj disease*).ti,ab,kw. 139035

5 exp Lung Diseases/ or (((lung* or pulmonary) adj3 disease*) or cystic fibrosis or asthma* or sarcoidos*).ti,ab,kw. 2092145

6 exp Neoplasms/ or (cancer* or neoplasm*).ti,ab,kw. 6166462

7 exp Diabetes Mellitus/ or (diabetes or diabetic or hyperglycemia* or glucose intolerance).ti,ab,kw. 1470584

8 exp Mental Disorders/ or (eating disorder* or anorexia nervosa or bulimi* or binge eat* or (self adj (injur* or mutilat*)) or suicide* or suicidal or parasuicid* or mood disorder* or affective disorder* or bipolar or mania or manic or depression or depressive or dysthymi* or neurotic or neurosis or adjustment disorder* or anxiety disorder* or obsess* or compulsi* or panic or phobi* or ptsd or posttrauma* or post trauma* or chronic fatigue* or affective symptoms or mental disorder* or mental health or schizo* or (mental* adj2 ill*)).ti,ab,kw. 3099444

9 2 or 3 or 4 or 5 or 6 or 7 or 8 15947740

10 *Cost-Benefit Analysis/ or *Health Care Cost/ or *Health economics/ or *Economic Evaluation/ or *Cost-Effectiveness Analysis/ or *Cost-minimization analysis/ or *"Costs and Cost Analysis"/ or ((economic or cost comparison or cost-effectiveness or cost-utility or cost-consequence* or cost-benefit or cost-minimi?ation or Combined intervention cost* or health economic* or health care cost or health* cost or medical cost) adj2 (evaluation* or analys* or reduc* or saving* or efficienc* or stud*)).ti,ab,kw. 184334

11 1 and 9 and 10 2281

12 Letter/ or Comment/ or Editorial/ 1960163

13 11 not 12 2266

14 Protocol/ 398

15 13 not 14 2266 (eksportert til EndNote)

**Ovid MEDLINE(R) and Epub Ahead of Print, In-Process, In-Data-Review & Other Non-Indexed Citations, Daily and Versions <1946 to October 03, 2023>**

1 *Telemedicine/ or *Telenursing/ or Mobile Applications/ or Smartphone/ or (telemedicine or tele-medicine* or telehealth or tele-health or telecare or tele-care or telehealthcare or tele-healthcare or telehome* or tele-home* or telemonitoring or tele-monitoring or telenurs* or tele-nurs* or telesupport* or tele-support* or telemental* or tele-mental* or telesurveillance or tele-surveillance or telefollow* or tele-follow* or telepatient* or tele-patient* or telemanag* or tele-manag* or e-care or ecare or e-health or ehealth or m-health or mhealth or mobile health or m-care or mcare or e-mental* or emental* or m-mental or mmental or telepsychiatry or tele-psychiatry or ((home or remote or digital) adj3 monitoring) or ((remote or distant or distance* or tele*) adj3 (care or healthcare or patient*)) or ((mobile or smartphone or tablet) adj3 (app or apps or application*)) or (iphone* or ipad* or smart device* or smartphone* or smart-phone* or web-based or online)).ti,ab,kw. 375928

2 Multiple Chronic Conditions/ or Multimorbidity/ or Comorbidity/ or Chronic disease/ or (multimorbid* or complex-care or multiple-condition? or multiple-chronic-condition? or chronically ill or ((long-term or chronic or co-morbid) adj (condition* or disease* or diagnosis* or illness*)) or (chronic adj (comorbidit* or co-morbidit*))).ti,ab,kw. 513377

3 exp Cardiovascular Diseases/ or (cardio* or cardia* or heart* or coronary* or angina* or ventric* or myocard* or pericard* or isch?m* or emboli* or arrhythmi* or thrombo* or atrial fibrillat* or tachycardi* or endocardi* or sick sinus or hypertension* or stroke or ((vascular or cerebrovascular or arterial) adj (disease* or disorder*)) or ventricular).ti,ab,kw. 4372896

4 Noncommunicable Diseases/ or ((non-communicable or non-infectious or noncommunicable or noninfectious or chronic) adj disease*).ti,ab,kw. 100656

5 exp Lung Diseases/ or (((lung* or pulmonary) adj3 disease*) or cystic fibrosis or asthma* or sarcoidos*).ti,ab,kw. 1375939

6 exp Neoplasms/ or (cancer* or neoplasm*).ti,ab,kw. 4557004

7 exp Diabetes Mellitus/ or (diabetes or diabetic or hyperglycemia* or glucose intolerance).ti,ab,kw. 863115

8 exp Mental Disorders/ or (eating disorder* or anorexia nervosa or bulimi* or binge eat* or (self adj (injur* or mutilat*)) or suicide* or suicidal or parasuicid* or mood disorder* or affective disorder* or bipolar or mania or manic or depression or depressive or dysthymi* or neurotic or neurosis or adjustment disorder* or anxiety disorder* or obsess* or compulsi* or panic or phobi* or ptsd or posttrauma* or post trauma* or chronic fatigue* or affective symptoms or mental disorder* or mental health or schizo* or (mental* adj2 ill*)).ti,ab,kw. 2059187

9 2 or 3 or 4 or 5 or 6 or 7 or 8 11852644

10 *Cost-Benefit Analysis/ or *Health Care Cost/ or *Health economics/ or *Economic Evaluation/ or *Cost-Effectiveness Analysis/ or *Cost-minimization analysis/ or *"Costs and Cost Analysis"/ or ((economic or cost comparison or cost-effectiveness or cost-utility or cost-consequence* or cost-benefit or cost-minimi?ation or Combined intervention cost* or health economic* or health care cost or health* cost or medical cost) adj2 (evaluation* or analys* or reduc* or saving* or efficienc* or stud*)).ti,ab,kw. 86215

11 1 and 9 and 10 1352

12 Letter/ or Comment/ or Editorial/ 2194255

13 11 not 12 1343

14 Protocol/ 0

15 13 not 14 1343 (overført til Endnote)


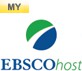


Wednesday, October 04, 2023 7:59:14 AM

**# Query Limiters/Expanders Last Run Via Results**

S8 ((MM "Cost Benefit Analysis" OR MM "Cost Effectiveness Analysis" OR MM "Costs and Cost Analysis" OR MM "Health Care Costs" OR TI(((economic or “cost comparison” or “cost- effectiveness” or “cost-utility” or “cost- consequence*” or “cost-benefit” or “cost-minimi?ation” or “Combined intervention cost*” or “health economic*” or “health care cost” or “health* cost” or “medical cost”) N1 (evaluation* or analys* or reduc* or saving* or efficienc* or stud*)) OR AB(((economic or “cost comparison” or “cost-effectiveness” or “cost-utility” or “cost-consequence*” or “cost-benefit” or “cost-minimi?ation” or “Combined intervention cost*” or “health economic*” or “health care cost” or “health* cost” or “medical cost”) N1 (evaluation* or analys* or reduc* or saving* or efficienc* or stud*))))) AND (S5 AND S6 AND S7)

Expanders - Apply equivalent subjects Search modes - Boolean/Phrase

Interface - EBSCOhost Research Databases Search Screen - Advanced Search Database - CINAHL

646

S7 (MM "Cost Benefit Analysis" OR MM "Cost Effectiveness Analysis" OR MM "Costs and Cost Analysis" OR MM "Health Care Costs" OR TI(((economic or “cost comparison” or “cost- effectiveness” or “cost-utility” or “cost- consequence*” or “cost-benefit” or “cost-minimi?ation” or “Combined intervention cost*” or “health economic*” or “health care cost” or “health* cost” or “medical cost”) N1 (evaluation* or analys* or reduc* or saving* or efficienc* or stud*)) OR AB(((economic or “cost comparison” or “cost-effectiveness” or “cost-utility” or “cost-consequence*” or “cost-benefit” or “cost-minimi?ation” or “Combined intervention cost*” or “health economic*” or “health care cost” or “health* cost” or “medical cost”) N1 (evaluation* or analys* or reduc* or saving* or efficienc* or stud*))))

Expanders - Apply equivalent subjects Search modes - Boolean/Phrase

Interface - EBSCOhost Research Databases Search Screen - Advanced Search Database - CINAHL

Display

S6 (MM "Telemedicine" OR MM "Telehealth" OR MM "Telenursing" OR MH "Mobile Applications" OR MH "Smartphone" OR TI (telemedicine or “tele-medicine*” or telehealth or “tele- health” or telecare or “tele-care” or telehealthcare or “tele-healthcare” or telehome* or “tele-home*” or telemonitoring or “tele-monitoring" or telenurs* or “tele-nurs*” or telesupport* or “tele-support*” or telemental* or “tele-mental*” or telesurveillance or “tele-surveillance” or telefollow* or “tele-follow*” or telepatient* or “”tele-

patient* or telemanag* or “tele-manag*” or “e-care” or ecare or “e-health” or ehealth or “m-health” or mhealth or

Expanders - Apply equivalent subjects Search modes - Boolean/Phrase

Interface - EBSCOhost Research Databases Search Screen - Advanced Search Database - CINAHL

Display

“mobile health” or “m-care” or mcare or “e-mental*” or emental* or “m-mental” or mmental or telepsychiatry or “tele- psychiatry” OR ((home or remote or digital) N2 (monitoring)) OR ((remote or distant or distance* or tele*) N2 (care or healthcare or patient*)) OR ((mobile or smartphone or tablet) N2 (app or apps or application*)) OR iphone* or ipad* or “smart device*” or smartphone* or “smart-phone*” or

“web-based” or online) OR AB

(telemedicine or “tele-medicine*” or telehealth or “tele-health” or telecare or “tele-care” or telehealthcare or “tele- healthcare” or telehome* or “tele- home*” or telemonitoring or “tele- monitoring" or telenurs* or “tele-nurs*” or telesupport* or “tele-support*” or telemental* or “tele-mental*” or telesurveillance or “tele-surveillance” or telefollow* or “tele-follow*” or telepatient* or “”tele-patient* or telemanag* or “tele-manag*” or “e-care” or ecare or “e-health” or ehealth or “m- health” or mhealth or “mobile health” or “m-care” or mcare or “e-mental*” or emental* or “m-mental” or mmental or telepsychiatry or “tele-psychiatry” OR ((home or remote or digital) N2 (monitoring)) OR ((remote or distant or distance* or tele*) N2 (care or healthcare or patient*)) OR ((mobile or smartphone or tablet) N2 (app or apps or application*)) OR iphone* or ipad* or “smart device*” or smartphone* or “smart-phone*” or “web-based” or online))

S5 ( (MH "Comorbidity" OR MH "Chronic Disease" OR TI(multimorbid* or multi- morbid* or”complex-care” or “multiple- chronic-condition*” or “multiple- condition*” or “chronically ill” OR ((“long-term” or chronic or “co-morbid”) W2 (condition* or disease* or diagnosis* or illness*)) OR ((chronic) W2 (comorbidit* OR “co-morbidit*”))) OR AB(multimorbid* or multi-morbid* or”complex-care” or “multiple-chronic- condition*” or “multiple-condition*” or “chronically ill” OR ((“long-term” or chronic or “co-morbid”) W2 (condition* or disease* or diagnosis* or illness*)) OR ((chronic) W2 (comorbidit* OR “co- morbidit*”)))) ) OR ( (MH "Cardiovascular Diseases" OR TI(cardio* or cardia* or heart* or coronary* or angina* or ventric* or myocard* or pericard* or isch?m* or emboli* or arrhythmi* or thrombo* or “atrial fibrillat*” or tachycardi* or endocardi* or “sick sinus” or hypertension* or stroke or ventricular OR ((vascular or cerebrovascular or

arterial) W1 (disease* or disorder*)) OR AB(cardio* or cardia* or heart* or coronary* or angina* or ventric* or myocard* or pericard* or isch?m* or emboli* or arrhythmi* or thrombo* or “atrial fibrillat*” or tachycardi* or

Expanders - Apply equivalent subjects Search modes - Boolean/Phrase

Interface - EBSCOhost Research Databases Search Screen - Advanced Search Database - CINAHL

Display

endocardi* or “sick sinus” or hypertension* or stroke or ventricular OR ((vascular or cerebrovascular or arterial) W1 (disease* or disorder*)))) ) OR ( ((MH "Noncommunicable Diseases") or TI(((“non-communicable” or “non-infectious” or noncommunicable or noninfectious or chronic) W1 (disease*)) or AB(((“non- communicable” or “non-infectious” or noncommunicable or noninfectious or chronic) W1 (disease*)))) ) OR ( (MH "Lung Diseases" OR TI(((lung* or pulmonary) N2 (disease*)) OR “cystic fibrosis” or asthma* or sarcoidos*) OR AB(((lung* or pulmonary) N2 (disease*)) OR “cystic fibrosis” or asthma* or sarcoidos*)) ) OR ( ((MH "Neoplasms") or TI(cancer* or neoplasm*) OR AB(cancer* or neoplasm*)) ) OR ( (MH "Diabetes Mellitus" OR TI(diabetes or diabetic or hyperglycemia* or “glucose intolerance”) OR AB(diabetes or diabetic or hyperglycemia* or “glucose intolerance”)) ) OR ( ((MH "Mental Disorders") or TI(“eating disorder*” or “anorexia nervosa” or bulimi* or “binge eat*” or (self W1 (injur* or mutilat*)) or suicide* or suicidal or parasuicid* or “mood disorder*” or “affective disorder*” or bipolar or mania or manic or depression or depressive or dysthymi* or neurotic or neurosis or “adjustment disorder*” or “anxiety disorder*” or obsess* or compulsi* or panic or phobi* or ptsd or posttrauma* or “post trauma*” or “chronic fatigue*” or “affective symptoms” or “mental disorder*” or “mental health” or schizo* or (mental* N1 ill*)) OR AB(“eating disorder*” or “anorexia nervosa” or bulimi* or “binge eat*” or (self W1 (injur* or mutilat*)) or suicide* or suicidal or parasuicid* or “mood disorder*” or “affective disorder*” or bipolar or mania or manic or depression or depressive or dysthymi* or neurotic or neurosis or “adjustment disorder*” or “anxiety disorder*” or obsess* or compulsi* or panic or phobi* or ptsd or posttrauma* or “post trauma*” or “chronic fatigue*” or “affective symptoms” or “mental disorder*” or “mental health” or schizo* or (mental* N1 ill*))) )

S4 ((MM "Cost Benefit Analysis" OR MM "Cost Effectiveness Analysis" OR MM "Costs and Cost Analysis" OR MM "Health Care Costs" OR TI(((economic or “cost comparison” or “cost- effectiveness” or “cost-utility” or “cost- consequence*” or “cost-benefit” or “cost-minimi?ation” or “Combined intervention cost*” or “health economic*” or “health care cost” or “health* cost” or “medical cost”) N1 (evaluation* or analys* or reduc* or saving* or efficienc* or stud*)) OR AB(((economic or “cost comparison” or

Expanders - Apply equivalent subjects Search modes - Boolean/Phrase

Interface - EBSCOhost Research Databases Search Screen - Advanced Search Database - CINAHL

646

“cost-effectiveness” or “cost-utility” or “cost-consequence*” or “cost-benefit” or “cost-minimi?ation” or “Combined intervention cost*” or “health economic*” or “health care cost” or “health* cost” or “medical cost”) N1 (evaluation* or analys* or reduc* or saving* or efficienc* or stud*))))) AND (S1 AND S2 AND S3)

S3 (MM "Cost Benefit Analysis" OR MM "Cost Effectiveness Analysis" OR MM "Costs and Cost Analysis" OR MM "Health Care Costs" OR TI(((economic or “cost comparison” or “cost- effectiveness” or “cost-utility” or “cost- consequence*” or “cost-benefit” or “cost-minimi?ation” or “Combined intervention cost*” or “health economic*” or “health care cost” or “health* cost” or “medical cost”) N1 (evaluation* or analys* or reduc* or saving* or efficienc* or stud*)) OR AB(((economic or “cost comparison” or “cost-effectiveness” or “cost-utility” or “cost-consequence*” or “cost-benefit” or “cost-minimi?ation” or “Combined intervention cost*” or “health economic*” or “health care cost” or “health* cost” or “medical cost”) N1 (evaluation* or analys* or reduc* or saving* or efficienc* or stud*))))

Expanders - Apply equivalent subjects Search modes - Boolean/Phrase

Interface - EBSCOhost Research Databases Search Screen - Advanced Search Database - CINAHL

54,856

S2 (MM "Telemedicine" OR MM "Telehealth" OR MM "Telenursing" OR MH "Mobile Applications" OR MH "Smartphone" OR TI (telemedicine or “tele-medicine*” or telehealth or “tele- health” or telecare or “tele-care” or telehealthcare or “tele-healthcare” or telehome* or “tele-home*” or telemonitoring or “tele-monitoring" or telenurs* or “tele-nurs*” or telesupport* or “tele-support*” or telemental* or “tele-mental*” or telesurveillance or “tele-surveillance” or telefollow* or “tele-follow*” or telepatient* or “”tele-

patient* or telemanag* or “tele-manag*” or “e-care” or ecare or “e-health” or ehealth or “m-health” or mhealth or “mobile health” or “m-care” or mcare or “e-mental*” or emental* or “m-mental” or mmental or telepsychiatry or “tele- psychiatry” OR ((home or remote or digital) N2 (monitoring)) OR ((remote or distant or distance* or tele*) N2 (care or healthcare or patient*)) OR ((mobile or smartphone or tablet) N2 (app or apps or application*)) OR iphone* or ipad* or “smart device*” or smartphone* or “smart-phone*” or

“web-based” or online) OR AB

(telemedicine or “tele-medicine*” or telehealth or “tele-health” or telecare or “tele-care” or telehealthcare or “tele- healthcare” or telehome* or “tele- home*” or telemonitoring or “tele- monitoring" or telenurs* or “tele-nurs*” or telesupport* or “tele-support*” or telemental* or “tele-mental*” or telesurveillance or “tele-surveillance” or

Expanders - Apply equivalent subjects Search modes - Boolean/Phrase

Interface - EBSCOhost Research Databases Search Screen - Advanced Search Database - CINAHL

180,555

telefollow* or “tele-follow*” or telepatient* or “”tele-patient* or telemanag* or “tele-manag*” or “e-care” or ecare or “e-health” or ehealth or “m- health” or mhealth or “mobile health” or “m-care” or mcare or “e-mental*” or emental* or “m-mental” or mmental or telepsychiatry or “tele-psychiatry” OR ((home or remote or digital) N2 (monitoring)) OR ((remote or distant or distance* or tele*) N2 (care or healthcare or patient*)) OR ((mobile or smartphone or tablet) N2 (app or apps or application*)) OR iphone* or ipad* or “smart device*” or smartphone* or “smart-phone*” or “web-based” or online))

S1 ( (MH "Comorbidity" OR MH "Chronic Disease" OR TI(multimorbid* or multi- morbid* or”complex-care” or “multiple- chronic-condition*” or “multiple- condition*” or “chronically ill” OR ((“long-term” or chronic or “co-morbid”) W2 (condition* or disease* or diagnosis* or illness*)) OR ((chronic) W2 (comorbidit* OR “co-morbidit*”))) OR AB(multimorbid* or multi-morbid* or”complex-care” or “multiple-chronic- condition*” or “multiple-condition*” or “chronically ill” OR ((“long-term” or chronic or “co-morbid”) W2 (condition* or disease* or diagnosis* or illness*)) OR ((chronic) W2 (comorbidit* OR “co- morbidit*”)))) ) OR ( (MH "Cardiovascular Diseases" OR TI(cardio* or cardia* or heart* or coronary* or angina* or ventric* or myocard* or pericard* or isch?m* or emboli* or arrhythmi* or thrombo* or “atrial fibrillat*” or tachycardi* or endocardi* or “sick sinus” or hypertension* or stroke or ventricular OR ((vascular or cerebrovascular or

arterial) W1 (disease* or disorder*)) OR AB(cardio* or cardia* or heart* or coronary* or angina* or ventric* or myocard* or pericard* or isch?m* or emboli* or arrhythmi* or thrombo* or “atrial fibrillat*” or tachycardi* or endocardi* or “sick sinus” or hypertension* or stroke or ventricular OR ((vascular or cerebrovascular or arterial) W1 (disease* or disorder*)))) ) OR ( ((MH "Noncommunicable Diseases") or TI(((“non-communicable” or “non-infectious” or noncommunicable or noninfectious or chronic) W1 (disease*)) or AB(((“non- communicable” or “non-infectious” or noncommunicable or noninfectious or chronic) W1 (disease*)))) ) OR ( (MH "Lung Diseases" OR TI(((lung* or pulmonary) N2 (disease*)) OR “cystic fibrosis” or asthma* or sarcoidos*) OR AB(((lung* or pulmonary) N2 (disease*)) OR “cystic fibrosis” or asthma* or sarcoidos*)) ) OR ( ((MH "Neoplasms") or TI(cancer* or neoplasm*) OR AB(cancer* or neoplasm*)) ) OR ( (MH "Diabetes

Expanders - Apply equivalent subjects Search modes - Boolean/Phrase

Interface - EBSCOhost Research Databases Search Screen - Advanced Search Database - CINAHL

2,144,296

Mellitus" OR TI(diabetes or diabetic or hyperglycemia* or “glucose intolerance”) OR AB(diabetes or diabetic or hyperglycemia* or “glucose intolerance”)) ) OR ( ((MH "Mental Disorders") or TI(“eating disorder*” or “anorexia nervosa” or bulimi* or “binge eat*” or (self W1 (injur* or mutilat*)) or suicide* or suicidal or parasuicid* or “mood disorder*” or “affective disorder*” or bipolar or mania or manic or depression or depressive or dysthymi* or neurotic or neurosis or “adjustment disorder*” or “anxiety disorder*” or obsess* or compulsi* or panic or phobi* or ptsd or posttrauma* or “post trauma*” or “chronic fatigue*” or “affective symptoms” or “mental disorder*” or “mental health” or schizo* or (mental* N1 ill*)) OR AB(“eating disorder*” or “anorexia nervosa” or bulimi* or “binge eat*” or (self W1 (injur* or mutilat*)) or suicide* or suicidal or parasuicid* or “mood disorder*” or “affective disorder*” or bipolar or mania or manic or depression or depressive or dysthymi* or neurotic or neurosis or “adjustment disorder*” or “anxiety disorder*” or obsess* or compulsi* or panic or phobi* or ptsd or posttrauma* or “post trauma*” or “chronic fatigue*” or “affective symptoms” or “mental disorder*” or “mental health” or schizo* or (mental* N1 ill*))) )

**Web of Science Date Run: Wed Oct 04 2023**

***The following Web of Science Core Collections searches where performed, this includes***

***- WOS.SCI: 1900 to 2023 (Science Citation Index)***

***- WOS.AHCI: 1975 to 2023 (Arts & Humanities Citation Index)***

***- WOS.ESCI: 2018 to 2023 (Emerging Sources Citation Index)***

***- WOS.ISTP: 1990 to 2023 (Conference Proceedings Citation Index – Science)***

***- WOS.SSCI: 1900 to 2023 (Social Sciences Citation Index)***

***- WOS.ISSHP: 1990 to 2023 (Conference Proceedings Citation Index – Social Sciences & Humanities)***

1: TS=(telemedicine or “tele-medicine*” or telehealth or “tele-health” or telecare or “tele-care” or telehealthcare or “tele-healthcare” or telehome* or “tele-home*” or telemonitoring or

“tele-monitoring” or telenurs* or “tele-nurs*” or telesupport* or “tele-support*” or telemental* or “tele-mental*” or telesurveillance or “tele-surveillance” or telefollow* or “tele-follow*” or telepatient* or “tele-patient*” or telemanag* or “tele-manag*” or “e-care” or ecare or “e-health” or ehealth or “m-health” or mhealth or “mobile health” or “m-care” or mcare or “e-mental*” or emental* or “m-mental” or mmental or telepsychiatry or “tele-psychiatry” or ((home or remote or digital) NEAR/2 (monitoring)) or ((remote or distant or distance* or tele*) NEAR/2 (care or healthcare or patient*)) or ((mobile or smartphone or tablet) NEAR/2 (app or apps or application*)) or (iphone* or ipad* or “smart device*” or smartphone* or “smart-phone*” or

“web-based” or online)) Results: 937038

2: TS=(multimorbid* or “complex-care” or “multiple-condition?” or “multiple-chronic-condition?” or “chronically ill” OR ((“long-term” or chronic or “co-morbid”) NEAR/0 (condition* or disease* or

diagnosis* or illness*)) or ((chronic) NEAR/0 (comorbidit* or “co-morbidit*”)))

Results: 164065

3: TS=(cardio* or cardia* or heart* or coronary* or angina* or ventric* or myocard* or pericard* or isch?m* or emboli* or arrhythmi* or thrombo* or “atrial fibrillat*” or tachycardi* or endocardi* or “sick sinus” or hypertension* or stroke or ((vascular or cerebrovascular or arterial) NEAR/0

(disease* or disorder*)) or ventricular) Results: 4256292

4: TS=((“non-communicable” or “non-infectious” or noncommunicable or noninfectious or chronic) NEAR/0 disease*) Results: 114592

5: TS=(((lung* or pulmonary) NEAR/2 disease*) or “cystic fibrosis” or asthma* or

sarcoidos*) Results: 486976

6: TS=(cancer* or neoplasm*) Results: 3273793

7: TS=(diabetes or diabetic or hyperglycemia* or “glucose intolerance”) Results: 955419

8: TS=((“eating disorder*” or “anorexia nervosa” or bulimi* or “binge eat*” or (self NEAR/0 (injur* or mutilat*)) or suicide* or suicidal or parasuicid* or “mood disorder*” or “affective disorder*” or bipolar or mania or manic or depression or depressive or dysthymi* or neurotic or neurosis or “adjustment disorder*” or “anxiety disorder*” or obsess* or compulsi* or panic or phobi* or ptsd or posttrauma* or “post trauma*” or “chronic fatigue*” or “affective symptoms” or “mental

disorder*” or “mental health” or schizo* or (mental* NEAR/1 ill*))) Results: 1588296

9: #8 OR #7 OR #6 OR #5 OR #4 OR #3 OR #2

Results: 9826859

10: TS=((economic or “cost comparison” or “cost-effectiveness” or “cost-utility” or

“cost-consequence*” or “cost-benefit” or “cost-minimization” or “cost-minimisation” or “Combined intervention cost*” or “health economic*” or “health care cost” or “health* cost” or “medical cost”)

NEAR/1 (evaluation* or analys* or reduc* or saving* or efficienc* or stud*)) Results: 151525

11: #1 AND #9 AND #10

Results: 1258

12: #1 AND #9 AND #10 and Article or Review Article or Proceeding Paper or Early Access

(Document Types) Results: 1218
