# Supplementary material for: Economic Evaluation Methodologies of Remote Patient Monitoring for Chronic Conditions: Scoping Review
Source: J Med Internet Res. 2025 Jul 4;27:e71565. doi: 10.2196/71565 (PMC12248258; doi:10.2196/71565)
Supplement: Multimedia Appendix 2 [file jmir-v27-e71565-s002.docx]

| Author, year | Cost categories | Cost measurement | Cost valuation |
| --- | --- | --- | --- |
| Achelrod D. et al., 2017 [34,35] | - Inpatient and outpatient treatment  - Pharmaceuticals  - Rehabilitation | - AOK Bayern (sickness fund) | - AOK Bayern (sickness fund) |
| Apantaku G. et al., 2022 [36] | - Health care utilization: visits to GP, specialist and ED, hospital admissions and length of stay - Out-of-pocket costs: professional household care, personal care, physiotherapy, etc. | - Patients' self-report | - Standard costs from different national sources |
| Blum and Gottlieb, 2014  [37] | - Number and days of hospitalisation - ED visits | - Medicare claims and payment | - Medicare payment |
| Carter H. E. et al., 2023 [38] | - Health service use: ED, hospital admissions and length of stay, and outpatient visits - Service delivery: labour, equipment and non-labour over-heads | - Administrative databases - MeCare programme | - Administrative databases |
| Chen Y.H. et al., 2013 [39] | - Health care use: inpatient, outpatient and ED | - Multiple hospital sources (details not reported) | - Multiple hospital sources (details not reported) |
| Clarke M. et al., 2018 [40] | - Health service resources: Hospitalisation, A&E, ambulance usage and home visits - Program: equipment, start-up and administration | - Multiple sources (details not reported) | - NHSD (National Health service Direct) and Primary Care Trusts 1&2 |
| Comin-Colet J. et al., 2016 [41] | - Health care use: Pharmacy, complementary examinations, referrals, outpatient care, emergency room visits, readmissions and procedures | - Hospital and primary care electronic medical records - Patient and caregiver interview | - Hospital cost accounting system (clinical activity-based costing) |
| De Batlle J. et al., 2021  [42] | - Health care resources: hospital admissions, ER visits, visits to specialists and primary care | - Electronic medical record | - Catalan Institute of Health official pricing |
| Deng L., 2015 [43] | - Patient travel and time use: number of visits to medical services, transportation, parking, time off from work | - Patient reported health care use | - Patient reported costs |
| Esteban C. et al., 2021 [44] | - Health care use: primary care visits, visits to the specialist and ED, and days admitted | - Department of analytical accounting | - Department of analytical accounting |
| Finkelstein S. M. et al., 2006 [45] | - Virtual visits: virtual visit time, nursing personnel compensation, equipment, technical support, administrative overhead | - Acquired equipment | - Internal revenue service mileage reimbursement - Average nurse compensation - Administrative overhead - Acquisition costs - Retail price |
| Frederix I. et al., 2019 [46] | - Health care use: readmission, cardiologist and nephrologist follow-up visits, and diagnostic tests | - Invoices (hospital financial department) | - Invoices (hospital financial department) |
| Greving J. P. et al., 2015 [47] | - Health care use: consultations, medication, inpatient hospital days - Patient travel: travel (km), parking, absence from paid and unpaid work - Intervention: staff time | - Patient reported health care visits - Electronic patient files - Time assessment | - Dutch costing manual - The Drug Information System of the Health Care Insurance Board - Medication price |
| Henderson C. et al., 2013 [48] | - Telehealth equipment incl. installation and maintenance, free phone numbers, licences - Telehealth support: staff time (patient monitoring), staff training, project management, back-office functions, administrative overheads - Health and social care use (incl. hospital, primary care, community services, drug treatment) | - Patient-reported service use - Information from each site | - Telehealth equipment prices and related expenditure - Top down and bottom-up calculation of telehealth monitoring staff time - Staffing costs incl. indirect costs (other staff) - National unit costs (health and social care) |
| Herold R. et al., 2018 [49] | - Health care use: in-patient, outpatient, medication, home health care, travel costs and rehabilitation | - Reimbursement data of the statutory health insurance | - Reimbursement data of the statutory health insurance |
| Ho Y. L. et al., 2014 [50] | - Health care use: medication, pharmacological service, examinations, diagnostic tests, physician visits, operations, anaesthesia, blood product, ward, nursing, and specialized equipment during visits to the OPD and ED, and hospitalisation. - Patients’ self-payment - Intervention: direct costs: staff costs, contact costs and fees (incl. telehealth equipment), and indirect costs: adm. costs, marketing and business development | - Electronic database at the hospital | - Administrative billing system |
| Inocencio T. et al., 2023 [51] | - Health care use: Inpatient, ED visits and medication | - Peer-reviewed literature - Direct evidence of use of the service | - Peer-reviewed literature adjusted to a Medicare payer population |
| Isaranuwatchai W. et al., 2018 [52] | - Health care utilization: hospitalisation and ER visits - Programme: personnel and supplies | - Alaya care mobile app (nurses and patients) | - Canadian Institute of Health Informatics |
| Jodar-Sanchez F. et al., 2014 [53] | - Health care utilization: A&E department visits, specialized care and hospital admissions - Professionals’ intervention: case manager time, clinical call centre time - Telehealth system: software and equipment, installation time | - The service supplying company (prices, time, travel) - Time estimations | - Andalusian Health Service (public prices, salary rates) - The service supplying company (software and equipment) |
| Lee J. K. et al., 2021 [54] | - Health care utilization: outpatient, ED and hospitalisation | - Medical records | - Electronic billing records |
| Maeng D. D. et al., 2014 [55] | - Health care utilization: hospitalisation | - Geisinger Health Plan claims data | - Geisinger Health Plan claims data |
| Mirón Rubio M. et al., 2018 [56] | - Health service use: telemonitoring nurse, hospital admission, ER, home hospitalisation, primary care visit, admission to intensive care unit | - Nurse scheduled working hours | - Publication by the Galician Health Service (reference prices) |
| Mudiyanselage S. B. et al., 2019 [57] | - Health care utilization: hospitalisation - Intervention: fixed and variable costs | - Hospital admission system | - Hospital costing system |
| Mudiyanselage S. B. et al., 2023 [58] | - Health care utilization: hospital admission - Intervention: software licence, protocol licence, project management and video conferencing, labour and travel | - Study team - Service data - Patients’ self-report | - DRG - Hospital LOS-information - Victorian Department of Health’s Weighted Inlier Equivalent Separation calculator - Unit prices (equipment) |
| Noel H. C. et al., 2004  [59] | - Healthcare use: bed-days of care, specialty and primary care visits, clinic visits, unscheduled clinic and ER visits and home visits | - Health provider's electronic database and home-base program - Community agencies - Distance between patients' home and healthcare provider | - Health provider's electronic database for resource utilisation - Transportation costs |
| Palmas W. et al., 2010 [60] | - Health care utilization: Inpatient services, physician services, outpatient services, durable medical equipment, home care, skilled nurse facilities and hospice services - Project intervention: vendors, bioinformatics teams (salary and equipment) and clinical teams (salary and equipment) | - Medicare claims - Vendors’ individual contracts - Project costs | - Medicare claims - Vendors’ individual contracts - Actual expenditures |
| Paré G. et al, 2006 [61] | - Health care utilization: home visits (time and transportation) and hospitalisations - Technology: licences, web phones, support, installation and maintenance | - Management control systems (logbooks) - Patient medical records | - Mean hourly rate set by collective agreement for graduate nurses - DRG - Market prices |
| Paré G. et al, 2013a  [62] | - Health services consumption: home visits, ER visits and hospitalisation associated with the primary diagnosis (number and length of stay) - Home telemonitoring: nurse time and travel costs - Technology: purchase and instalment, hosting and maintaining regional server, training, support | - Number and length of home visits - Negotiated prices with the application’s supplier | - Average hourly rate (nurses’ collective agreement) - Mileage rate - Negotiated prices with the application’s supplier |
| Paré G. et al, 2013b [63] | - Health care utilization: home visits, ER visits, hospitalisation - Intervention costs: - Home telemonitoring: nurse time and travel - Technology: purchases, installing, hosting and maintaining servers, professional services | - Computerised medical records - Information systems at the JR Health Centre - Scheduled time spent by nurses - Negotiated prices with the application’s supplier | - Average hourly rate (nurses’ collective agreement) - Mileage rate - Negotiated prices with the application’s supplier |
| Pathak A. et al., 2022 [64] | - Health care utilisation: hospitalisation HF (Hospitalisation, day-care, visits ED, drugs, general and HF hospital consultations), post hospitalisation expenditure HF (hospitalisation in post -acute care facility, home-based care) and ambulatory HF (drugs, personnel fees, lab. tests, medical procedures, transport) | - French national health insurance data system | - Official French national tariffs 2019 |
| Riley W. T. et al., 2015 [65] | - Health care utilisation: number and days of hospitalisation | - Hospital charges | - Hospital charges |
| Sohn S. et al., 2012 [66] | - Health care resources: Medication, sickness reimbursement, therapeutic aids, hospitalisation, rehabilitation/wellness | - The health insurance database | - The health insurance database |
| Stoddart A. et al., 2015 [67] | - Health care utilisation: number of consultations with GP and nurse, unscheduled care service, NHS24 and LUCS consultations, COPD medication, hospital admissions - Intervention: equipment, installation and maintenance, training of patients incl. travel, monitoring, alert handling | - Anecdotal descriptions from staff - Contracted services - Time sheets - Patient questionnaires - Previous surveys - Recordings made by nurses - Patients’ secondary care records | - Average hourly wage - Standard UK price weights - British National Formulary - Weighted averages in non-respiratory wards |
| Sydow H. et al., 2022 [68] | - Health care utilisation: cardiovascular hospitalisation, outpatient treatment, therapeutic appliances, health care products, rehabilitation, medications, home nursing care, transportation, sickness leave payment - Intervention: infrastructure, patient measuring devices, personnel | - Statutory health insurance claims data | - Statutory health insurance claims data |
| Vestergaard A. S. et al., 2020 [69] | - Health care utilisation: Pre-hospital services, in-patient and outpatient, municipality-based health and social care primary health care, prescribed medicines - Intervention: capital (development of telekit, telekit, start-up, training), and operation (maintenance, support, licences) | - National Health Insurance Register - Danish National Patient Register - Danish National Prescription Register - Registrations in municipalities - Licences - Project registration of intervention costs - Payment to supplier | - DRG - GP fees (quoted agreements) - Pharmacy selling prices - Average effective wages for nurses - Estimated prices for stays in nursing homes - Licences - Payment to supplier - Expected purchase price |
| Warren R. et al., 2018  [70] | - Health care utilisation: hospital admissions, specialist visits, number of visits to the GP  - Intervention: recruitment, set-up, health assessment, CC consultations, monitoring and triage, software maintenance, internet connection, communications with other health professionals and equipment removal | - Data collected by the research team on trial costs  - GPs and public hospitals | - DRG  - Actual trial costs |
| Willems D. C. M. et al., 2007 [71] | - Health care utilisation: hospital care, GPs and other health professionals, prescribed medication, home care - Intervention: materials, personnel, telephone and travel - Patient and family: over-the-counter medication, informal care - Productivity losses | - Hospital billing system - Patient cost diary - Time registration (nurses) - Data on trial set-up collected by the research team - Registration of nurses’ time | - Dutch manual for cost research - Ministry of Education, Culture and Science - Unit prices (medication and equipment) - Salary incl. overhead (nurses) |
| Witt Udsen F. et al., 2017a [72] | - Health care utilisation: admissions, outpatient, emergency wards visits, municipal standard care incl. home nursing care and rehabilitation, GP visits, prescribed medication - Intervention: hardware and peripherals, installation, maintenance, support, training for health care professionals, patient-specific training, monitoring, project management, IT infrastructure and application maintenance | - Danish National Patient Register - Danish Register for COPD - National Health Insurance Service Register - The Danish Register of Medical Product Statistics - Individual care systems in municipality districts (Type and duration of standard care) - Planned time for workshops | - DRG - Reference prices - Fees negotiated in a collective agreement - Standardised pharmacy consumer price - Average wages for health care professional and adm. officers - Prices paid of telehealthcare equipment, licences, maintenance and support - Negotiated prices of installation |
| Witt Udsen F. et al., 2017b [73] | - Ref. F. Witt Udsen et al., 2017a | Ref. F. Witt Udsen et al., 2017a | Ref. F. Witt Udsen et al., 2017a |
| Zaman S. et al., 2023  [74] | - Secondary health care use: emergency department attendances, unplanned admissions, elective admissions, cardiology outpatient attendances - Platform costs | - Electronic health record - The *Discover* data platform | - Details not reported |
| Ziegler A. et al., 2023 [75] | - Health care utilization: hospitalisation, ambulant, prescription medicine - Intervention: medical devices and software, infrastructure costs (office rent, computer system, workplace, telephone system) | - Individual cost data | - Health insurance companies - Actual labour costs - Prices of devices and software - Assumed infrastructure costs |
