# Supplementary material for: Economic Evaluation Methodologies of Remote Patient Monitoring for Chronic Conditions: Scoping Review
Source: J Med Internet Res. 2025 Jul 4;27:e71565. doi: 10.2196/71565 (PMC12248258; doi:10.2196/71565)
Supplement: Multimedia Appendix 3 [file jmir-v27-e71565-s003.docx]

Yes: No: Not applicable:
